# Alterations of GABAergic Signaling in Autism Spectrum Disorders

**DOI:** 10.1155/2011/297153

**Published:** 2011-06-23

**Authors:** Rocco Pizzarelli, Enrico Cherubini

**Affiliations:** Neurobiology Sector and IIT Unit, International School for Advanced Studies (SISSA), Via Bonomea 265, 34136 Trieste, Italy

## Abstract

Autism spectrum disorders (ASDs) comprise a heterogeneous group of pathological conditions, mainly of genetic origin, characterized by stereotyped behavior, marked impairment in verbal and nonverbal communication, social skills, and cognition. Interestingly, in a small number of cases, ASDs are associated with single mutations in genes encoding for neuroligin-neurexin families. These are adhesion molecules which, by regulating transsynaptic signaling, contribute to maintain a proper excitatory/inhibitory (E/I) balance at the network level. Furthermore, GABA, the main inhibitory neurotransmitter in adult life, at late embryonic/early postnatal stages has been shown to depolarize and excite targeted cell through an outwardly directed flux of chloride. The depolarizing action of GABA and associated calcium influx regulate a variety of developmental processes from cell migration and differentiation to synapse formation. Here, we summarize recent data concerning the functional role of GABA in building up and refining neuronal circuits early in development and the molecular mechanisms regulating the E/I balance. A dysfunction of the GABAergic signaling early in development leads to a severe E/I unbalance in neuronal circuits, a condition that may account for some of the behavioral deficits observed in ASD patients.

## 1. Introduction

Autism spectrum disorders (ASDs) comprise a complex and heterogeneous group of pathological conditions including autism, Rett and Asperger syndromes, and pervasive developmental disorder-otherwise nonspecified, characterized by impaired social interactions, deficits in verbal and nonverbal communication, and a limited interest in the surrounding environment associated with stereotyped and repetitive behaviors [1]. The incidence of these disorders, which varies between 10 and 20 per 10000 children, has risen dramatically over the past two decades mainly because of the use of broader diagnostic criteria and the increased attention of the medical community [2]. Clinical signs are usually present at the age of 3 years, but prospective studies of infants at risk have demonstrated that deficits in social responsiveness, communication, and play could be present already at the age of 6–12 months. 

ASDs are the most heritable neurodevelopmental disorders of early childhood. Genetic factors are thought to account for *∼*80% of autism cases, and since autism is a spectrum of disorders, it is conceivable that in most cases different genes act in combination in different individuals [3]. Genes, interacting with epigenetic factors, may influence neuronal migration, axon pathfinding, dendritic development, synaptogenesis, and pruning, thus contributing to alter neuronal connectivity and information processing [4]. 

Interestingly, a small percentage of ASDs patients carry single mutations in genes encoding for synaptic cell adhesion molecules of the neurexin (NRXN)-neuroligin (NLG) families [5]. These include mutations in genes encoding for NRXN1 [6, 7], for NLG3, NLG4 [8–10], and for Shank3 [11]. Although rare, these mutations provide crucial information on the synaptic abnormalities which possibly affect ASDs patients and point to synapses dysfunction as a possible site of autism origin. Synapses are specialized intercellular junctions which transfer information from a neuron to a target cell, usually another neuron. 

Several lines of evidence suggest that an impairment of GABAergic transmission contributes to the development of ASDs. GABA, the main inhibitory neurotransmitter in adulthood is released by interneurons which contain the GABA synthesizing enzymes glutamic acid decarboxylase (GAD)65 and GAD67. GABAergic interneurons, which constitute a heterogeneous group of cells, differently classified in virtue of their anatomical, physiological, and molecular features [12], represent only 10%–15% of the total neuronal population. Nevertheless, they provide the functional balance, complexity, and computational architecture of neuronal circuits [13]. They play a key role in regulating neuronal excitability *via* feedback and feed-forward inhibition. Axons of different inhibitory cells target different postsynaptic subcellular compartments, allowing them to selectively control the output of pyramidal cells [14], thus providing the temporal structure that orchestrates the activity of neuronal ensembles leading to coherent network oscillations [15]. 

While in the mature brain GABA acts as an inhibitory transmitter, during the embryonic and the perinatal period, this neurotransmitter depolarizes targeted cells and triggers calcium influx. GABA-mediated calcium signaling regulates a variety of different developmental processes from cell proliferation migration, differentiation, synapse maturation, and cell death [16]. Although the geometry and the cellular and subcellular selectivity of GABAergic axons are mainly genetically determined, axonal branching and arborization are regulated by activity and experience and often require brain-derived neurotropic factor (BDNF, [17]). Thus, sensory stimulation contributes to shape neuronal circuits, whereas sensory deprivation significantly retards their maturation [18–20].

Considering the multifacet of GABA activities particularly during development, it is not surprising that disturbance of GABAergic signaling can result in aberrant information processing, as found in neurodevelopmental disorders such as ASDs. In particular, it has been hypothesized that at least some forms of autism result from an imbalance between excitation and inhibition in local circuits involved in sensory, mnemonic, social, and emotional processes. The resulting hyperexcitability could disrupt the normal formation of cortical maps leading to a relatively unstable state [21]. The cortex is organized in vertical mini columns of functionally related glutamatergic and GABAergic neurons that process thalamic inputs. Local GABAergic circuits contribute to control the functional integrity of minicolumns *via* lateral inhibition. Interestingly, analysis of postmortem tissues from ASDs patients has revealed alterations in the number of mini columns, in the horizontal spacing separating cell columns, and in their internal structure [22]. The abnormal cytoarchitecture is often associated with an increased expression of calbindin-, calretinin- and parvalbumin-positive GABAergic interneurons [23]. In addition, changes in GAD65 and GAD67 [24], in the mRNA encoding for these enzymes [25–27], in GABA_A_ [28, 29] and GABA_B_ receptors [30] have been found in brain samples from ASDs patients. The altered GABAergic function may reduce the threshold for developing seizures as demonstrated by the high comorbidity of ASDs with epilepsy (one third of ASDs patients have seizures [31]). This further strengthens the hypothesis that an unbalance between excitation and inhibition contributes to these devastating neurological disorders. 

This paper will focus on the functional role of GABA in regulating developmental processes, their experience-dependent refinement and, at the network level, the balance between excitation and inhibition. In addition, the implications that an altered GABAergic signaling may have in neurodevelopmental disorders such as ASDs will be discussed taking into account different animal models.

## 2. GABA, a Pioneer Neurotransmitter in Neuronal Circuits Formation

**Figure 1:**
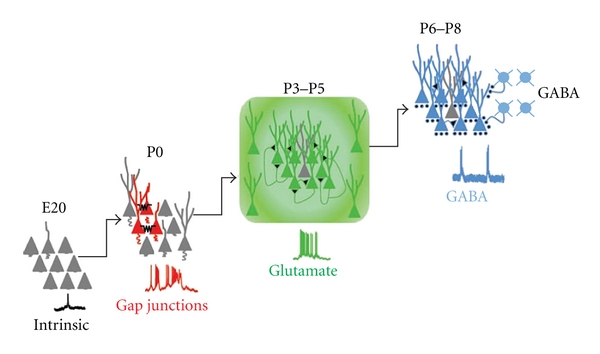
Patterns of electrical activity observed at late embryonic/early postnatal stages in the cortex. E20: uncorrelated calcium spikes; P0: Spontaneous Plateau Assemblies (SPAs) synchronized by gap junctions. P3–P5: early network oscillations (ENOs) mediated by glutamate. P6–P8: giant depolarizing potentials (GDPs) mediated by GABA and glutamate. (Modified from [32]).

The construction of the brain relies on a series of well-defined genetically and environmentally driven factors whose disruption leads to pathological disorders including ASDs. During central nervous system development, a sequence of temporally related events during which neurons proliferate, migrate, differentiate, and establish proper synaptic connections occurs [16]. Further refinement of immature networks needs adaptive processes involving experience- or activity-dependent mechanisms, which lead to the formation of new synapses and elimination of others. Using imaging techniques and electrophysiological approaches, several patterns of coherent activity have been characterized early in development [32]. Uncorrelated spontaneous activity consisting of calcium action potentials has been suggested to play a crucial role in regulation of cortical neurogenesis at late embryonic stages [16, 33]. At birth, synchronous neuronal activity can be detected in the hippocampus and in the neocortex. This relies firstly on the activation of intrinsic conductances and gap junctions and later on synapse-driven events. Thus, small cell assemblies coupled to gap junctions generate nonsynaptic spontaneous plateau assemblies (SPAs, [32], Figure 1). 

These involve small groups of neurons and are associated with sustained intrinsic membrane potential oscillations. SPAs are modulated by oxytocin, a maternal hormone essential for labour induction, which transiently converts GABA action from excitatory to inhibitory [34]. As the network matures and the density of functional synapses increases, synaptic-driven network oscillations replace SPAs. A downregulation in the expression of connexins *via* CREB signalling, following activation of NMDA receptors, may lead to SPAs silencing [35]. Two different patterns of network-driven synaptic oscillations have been described: the giant depolarizing potentials or GDPs [36] and early network oscillations or ENOs [37]. These are reminiscent of “long oscillations” and “spindle bursts”, respectively, recorded from the rat somatosensory cortex *in vivo* [38] or of discontinue activity patterns observed in the EEG of preterm babies [39]. While ENOs (which usually precede GDPs) were initially thought to constitute the cortical counterpart of hippocampal GDPs, they have been shown to coexist with GDPs in the neocortex [32]. In the neocortex, ENOs critically depend on the activation of NMDA receptors [37]. In addition, evidence has been provided that extrasynaptic NMDA receptors activated by ambient glutamate generate a tonic current, which contributes to depolarize the membrane, to enhance cell excitability and to convert silent synapses into functional ones [40]. The activation of NMDA receptors by “ambient” glutamate would be facilitated by changes in subunits composition [41], in voltage dependence of the magnesium block [42] and in the high affinity for glutamate of extrasynaptic NMDA receptors. 

In the hippocampus, GDPs are generated by the synergistic action of glutamate and GABA, which in the immediate postnatal period, orchestrates neuronal ensembles *via* its depolarizing and excitatory action [43]. Before synapses formation, GABA depolarizes targeted neurons *via* a paracrine type of action. GABA released in a calcium- and SNARE-independent way by nonconventional release sites such as growth cones and astrocytes diffuses away to activate extrasynaptic receptors [44]. The absence of an efficient uptake system will enable GABA to accumulate in the extracellular space and to reach a concentration sufficient to exert its distal action. The depolarizing action of GABA would activate voltage-dependent calcium channels and would facilitate the relief of the voltage-dependent magnesium block from NMDA receptors, thus allowing calcium entry and activation of second messengers. 

Using network dynamics imaging, online reconstruction of functional connectivity and targeted whole-cell recordings, it has been recently demonstrated that, in immature hippocampal slices, functional hubs composed of subpopulations of GABAergic interneurons with large axonal arborizations are able to synchronize large neuronal ensembles [45]. The depolarizing action of GABA in immature neurons results from an outwardly directed flux of chloride. Chloride homeostasis is controlled by the Na-K-2Cl cotransporter NKCC1 and by the K-Cl cotransporter KCC2 that enhance and lower [Cl^−^]_*i*_, respectively [46]. Due to the low expression of the KCC2 extruder at birth, chloride accumulates inside the neuron *via* NKCC1. The progressive increase in the expression of KCC2 is responsible for the developmental shift of GABA from the depolarizing to the hyperpolarizing direction. KCC2 extrudes K^+^ and Cl^−^ using the electrochemical gradient for K^+^. Cl^−^ extrusion is weak in immature neurons and increases with neuronal maturation. 

**Figure 2:**
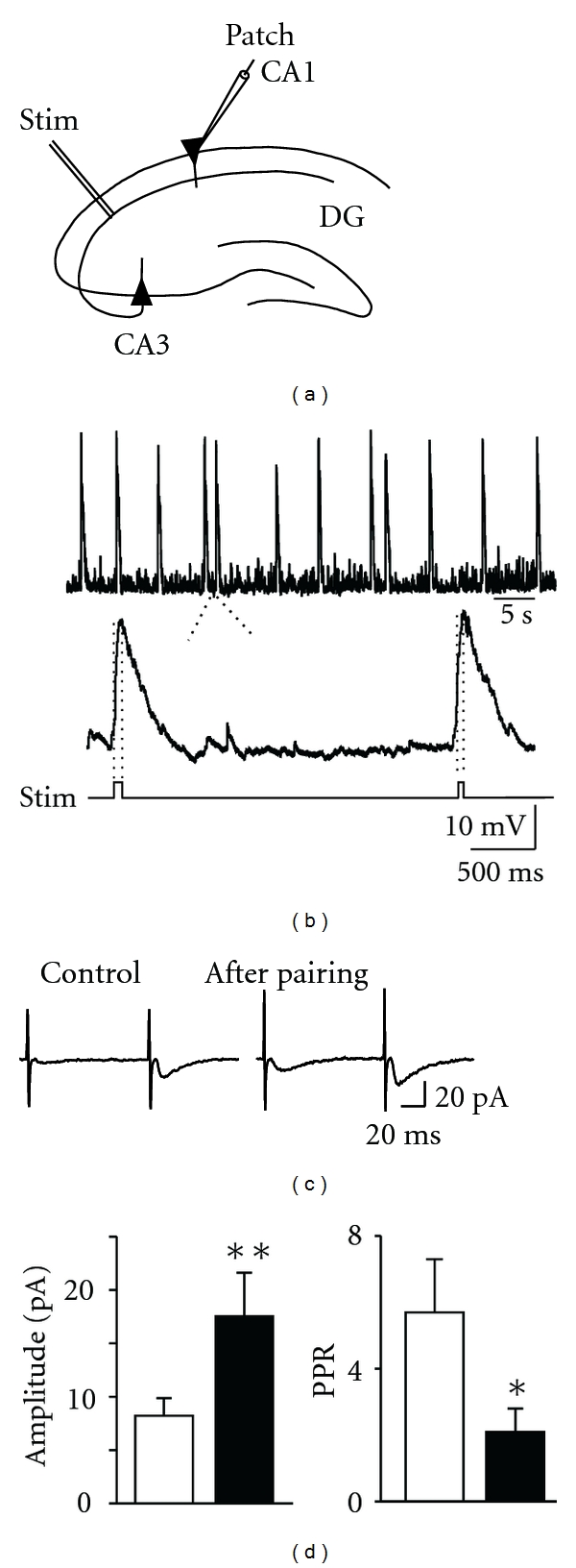
Pairing GABA-mediated GDPs with Schaffer collateral stimulation persistently enhances synaptic strength at glutamatergic CA3-CA1 connections. (a) Experimental paradigm. (b) The rising phase of GDPs (between the dashed lines) was used to trigger synaptic stimulation (stim) (c) EPSCs evoked in CA1 principal cells by minimal stimulation of Schaffer collateral, before and after pairing (average of 19 responses). (d) Each bar represents the mean peak amplitude of synaptic responses including failures (*n* = 8) and the paired pulse ratio (PPR; *n* = 8), obtained before (open) and after (closed) pairing. (Modified from [50]).

The functional role of the depolarizing action of GABA on early circuits development has been assessed by manipulating the expression levels of KCC2 and NKCC1, respectively. Thus, the premature expression of KCC2, has been shown to convert the action of GABA from excitatory to inhibitory and to impair the morphological maturation of cortical cells, without altering their radial migration [47]. This effect can be mimicked by overexpressing the inwardly rectifying K^+^ channel which lowers the membrane potential and reduces cell excitability, strongly suggesting that membrane depolarization caused by the early GABA excitation is essential for the functional maturation of cortical circuits *in vivo*. On the other hand, knocking down the expression of NKCC1 to abolish GABA_A_-mediated excitation, leads to a significant reduction in AMPA receptor-mediated synaptic transmission associated with a disruption of dendritic arborization and spines density further indicating that the depolarizing and excitatory action of GABA plays a permissive role in the formation of excitatory synapses [48]. Interestingly, these effects could be rescued by over expressing a mutant form of voltage-independent NMDA receptors, indicating that GABA depolarization cooperates with NMDA receptor to regulate the formation of excitatory synapses. It is worth noting that GDPs and associated calcium transients act as coincidence detectors for enhancing, in an associative type of manner, synaptic efficacy at emerging GABAergic [49], and glutamatergic synapses [50]. Using a “pairing” procedure, consisting of correlating GDPs-associated calcium rise with stimulation of mossy fibers or Schaffer collaterals, in the CA3 and CA1 region, respectively, we found that this procedure produced a strong and persistent potentiation of synaptic responses (Figure 2). 

In the absence of pairing, no significant changes in synaptic efficacy could be detected. Similar results were obtained by progressively increasing the interval between GDPs and mossy fiber/Schaffer collateral stimulation. Pairing-induced potentiation was prevented when the cells were loaded with the calcium chelator BAPTA or when nifedipine (but not the NMDA receptor antagonist D-(-)-2-amino-5-phosphonopentanoic acid) was added to the extracellular medium, suggesting that activity-dependent changes in synaptic efficacy depend on calcium rise through voltage-dependent calcium channels and not *via* NMDA receptors. 

Immature neurons are characterized by an elevated number of “silent” synapses [40]. These are synapses that do not conduct at rest either, because the neurotransmitter is not released when the presynaptic terminal is invaded by an action potential (presynaptically silent), or because they are unable to detect the release of the neurotransmitter due to the lack of the respective receptors on the subsynaptic membrane (postsynaptically silent). Silent synapses can be converted into active ones by activity-dependent processes and this represents the most common mechanism for LTP induction, not only during development but also in the mature brain [51]. Interestingly, the pairing procedure was able to convert silent synapses into active ones. In particular, in double pulse experiments, pairing caused the appearance of responses to the first stimulus and increased the number of successes to the second one, indicating that an increased probability of transmitter release accounts for long-term increase in synaptic strength. Therefore, calcium entry through voltage-dependent calcium channels, activated by the depolarizing action of GABA during GDPs, is instrumental in enhancing the number of functional GABAergic and glutamatergic synapses and/or the probability of GABA and glutamate release in a Hebbian way. This may contribute to refine neuronal connectivity before the establishment of the adult neuronal circuit. 

## 3. Molecular Determinants of GABAergic Synapses Formation

In the adult brain, information processing relies on the integration of excitatory and inhibitory circuits which use glutamate and GABA/glycine as neurotransmitters, respectively. The so-called excitatory/inhibitory (E/I) balance represents a critical condition for the correct functioning of neuronal networks and it is essential for nearly all brain functions, including representation of sensory information and cognitive processes. The E/I balance is maintained *via* highly regulated homeostatic mechanisms [52]. Neurons are able to compensate for experimental perturbations by modulating ion channels, receptors, signaling pathways, and neurotransmitters. At the molecular level, these processes require chromatin remodeling, changes in gene expression and repression, changes in protein synthesis, turnover and cytoskeleton rearrangement [53]. A disruption of the homeostatic control, due to the lack of compensatory changes, leads to an imbalanced E/I ratio and to the developmental of neuropsychiatric disorders including mental retardation, epilepsy and ASDs [21]. 

During brain maturation, the development of a proper E/I balance is achieved with the shift of GABA action from the depolarizing to the hyperpolarizing direction, a process that in rodents starts appearing toward the end of the first, beginning of the second postnatal week [54]. Disturbances in the E/I balance may also occur when the formation or maintenance of one class of synapses prevails over the others. The selective loss of excitatory or inhibitory synapses can take place during the initial period of synapse formation and consolidation or late in development during activity-dependent refinement of neuronal circuits and may involve mutations in genes encoding for ion channels or GABA_A_ receptor subunits. These would lead to circuits with abnormal activity and prone to seizures [55]. For example, the disruption in mice of the *gabrb3 *gene, which encodes for *β*3 subunits of GABA_A_ receptors, highly expressed during development, is sufficient to cause phenotypic traits which parallel those present in the Angelman syndrome [56]. Thus, mice lacking the *β*3 subunits exhibit a major reduction of GABA_A_ receptors, thalamic disinhibition and seizures associated with learning and memory deficits, poor motor skills on a repetitive task, hyperactivity, and a disturbed rest-activity cycle, all features characteristic of children affected by this neurological disorder. The cellular and molecular mechanisms underlying these phenomena are still poorly understood and their comprehension is further complicated by intrinsic differences among neuronal types, experimental conditions and the developmental stage of neurons [57]. 

During neuronal circuit assembly, GABA signaling precedes and promotes the formation of glutamatergic synapses [58]. The sequential development of GABA- and glutamate-mediated connections is independent on the arrival of afferent inputs but is related to the degree of maturation of targeted cells including changes in dendritic length, in somatic size and capacitance [58]. While functional GABAergic synapses require the presence of small apical dendrites in stratum radiatum of the hippocampus, glutamatergic connections require the presence of dendrites in stratum lacunosum moleculare. 

The refinement of GABAergic connections and their translation into a potent inhibitory network is a protracted process which extends well beyond the first two postnatal weeks into the adolescent period and is regulated by neuronal activity and experience. In the visual cortex, for instance, experience-dependent regulation of the GABAergic innervation controls the onset of critical periods [59] during which neuronal circuits display a heightened sensitivity to environmental stimuli and are greatly shaped by sensory experience. Thus, a delayed and an accelerated onset in visual plasticity can be obtained by negatively or positively interfering with the GABAergic function, respectively [59]. GABA signaling itself would be responsible for the development of inhibitory connections as demonstrated by the observation that, knocking down GAD67 in basket interneurons severely impairs GABAergic innervation [20]. These effects may be attributed to the activity-dependent reduction in GABA synthesis and signaling following down regulation of GAD67 levels and/or enzyme activity [20]. 

To be highly efficient, synaptic transmission requires the presence of clustered postsynaptic receptors localized in precise apposition to presynaptic release sites. At inhibitory connections, this task is accomplished by gephyrin, a tubulin-binding protein which traps glycine and GABA_A_ receptors in the right place anchoring them to the cytoskeleton [60].

**Figure 3:**
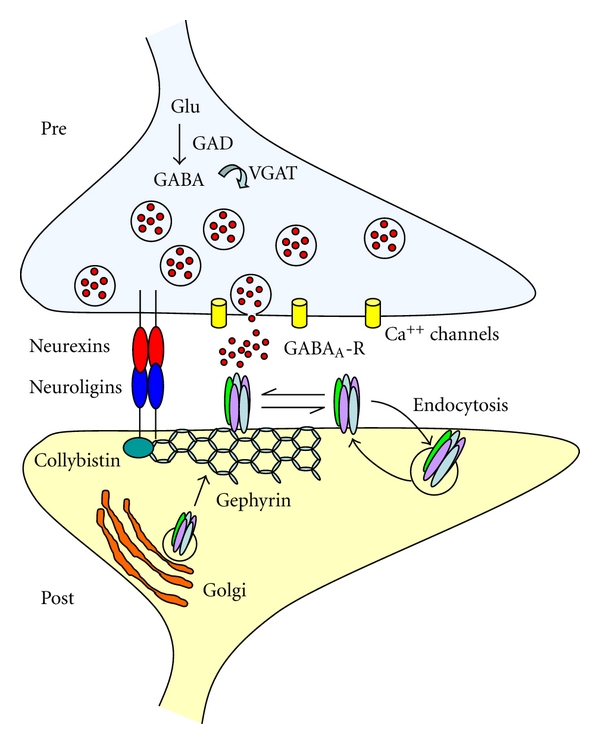
Structural organization of GABAergic synapses. The postsynaptic organization comprises a large number of proteins that allow the correct targeting, clustering and stabilization of GABA_A_ receptors. Among them gephyrin forms hexagonal lattices that trap GABA_A_ receptors in precise apposition to presynaptic release sites. Cell adhesion molecules of the neuroligin-neurexin families bridge the cleft and ensure transsynaptic signaling, essential for the maintenance of a proper E/I balance.

 Interestingly, a recent study has demonstrated that gephyrin directly interacts with adhesion molecules of the NLGs family [61] which in turn bind to their presynaptic partners NRXNs to regulate transmitter release (Figure 3). Therefore, gephyrin plays a key role not only in stabilizing GABA_A_ receptors but also in regulating transsynaptic signaling and in maintaining an appropriate E/I balance. The NLG-NRXN complexes possess a potent “synaptogenic” or synapses organizing activities as demonstrated by their ability to induce presynaptic differentiation of contacting neuritis when expressed in heterologous nonneuronal cells. Postsynaptic NLGs promote the assembly of functional presynaptic specializations in axons while presynaptic NRXNs recruit postsynaptic scaffolding proteins and neurotransmitter receptors in dendrites *via *their interaction with NLGs [62]. By functionally coupling synaptic calcium channels with the release machinery, NRXNs are thought to play an essential role in calcium-triggered neurotransmitters release [63]. The NLGs family comprises five different genes (NLG1-NLG5 with various splice variants), which form homodimers through the extracellular domain. Among these, NLG2 is preferentially associated with GABAergic synapses, while NLG1 with glutamatergic synapses [64, 65]. The NRXN family includes *α*- and *β*-NRXN. Initially, *β*-NRXN was considered the main partner for NLG, but recently, also *α*-NRXN was found to bind NLG [66]. Unlike *β*-NRXN that participates in the formation of both glutamatergic and GABAergic synapses, *α*-NRXN seems to be specific for GABAergic synapses [67]. Therefore, it is clear that within a neuronal network, the NLG-NRXN interaction controls the formation of both glutamatergic and GABAergic synapses [68]. At inhibitory synapses, GABA_A_ receptors are firstly assembled in the endoplasmic reticulum from appropriate subunits and then delivered to the plasma membrane. Targeting and clustering GABA_A_ receptors at synaptic and extrasynaptic sites is dynamically regulated by neuronal activity [69] and requires the precise interplay of various proteins and active transport processes along the cytoskeleton [60, 70]. 

Disrupting endogenous gephyrin with selective antibodies led to a reduction of GABA_A_ receptor clusters [71], an effect that was associated with a decrease in the density and size of NLG2 clusters and with a loss of GABAergic innervation (Kasap, personal communication). Thus, pair recordings from interconnected cells demonstrated that, respect to controls, neurons transfected with recombinant antibodies against gephyrin exhibited a lower probability of GABA release. This reduction likely involves NLG2 which is preferentially concentrated at inhibitory synapses and directly binds gephyrin through a conserved cytoplasmatic domain [61]. Similarly, at glutamatergic synapses, the NLG-NRXN complex has been shown to act as a coordinator between postsynaptic and presynaptic sites [72]. Hence, overexpressing the glutamatergic scaffold protein PSD-95 on the postsynaptic site enhanced the probability of glutamate release *via* a retrograde modulation of neurotransmitter release which probably involves the NLG-NRXN complex. From the reported data, it is not surprising that single mutations in genes encoding for adhesion molecules belonging to the NLG-NRXN families, such as those found in few cases of ASDs [73], lead to defective architectural structuring of synaptic connections, molecular assembly of synapses and an E/I unbalance.

As outlined in the next section, the use of animal models of ASDs has enabled to investigate the mechanistic basis of the E/I imbalance for a range of neurodevelopmental disorders. 

## 4. Altered GABAergic Signaling in Animal Models of ASDs

A dysfunction of GABAergic signaling mediates autism-like stereotypes in the majority of animal models of ASDs obtained by experimentally manipulating candidate genes for autism susceptibility or environmental risk factors. The characteristic ASDs phenotype is often associated with either a loss or a gain of the GABAergic function. Consistent with postmortem studies from brain tissues obtained from ASDs patients [74] alterations in GABA synthesising enzymes GAD65 and GAD67, in GABA release, in the expression of particular subtypes of GABA_A_ receptors have been described. 

A presynaptic reduction in glutamic acid decarboxylase 1 (*Gad1*) and glutamic acid decarboxylase 2 (*Gad2*) mRNA encoding for GAD67 and GAD65, respectively, has been recently found in mice lacking the *Mecp2* gene in GABA releasing neurons (*Viaat-Mecp2^−/y^*, [75]). Mutations in the X-linked *Mecp2* gene, which encodes the transcriptional regulator methyl-CpG-binding protein 2 (MeCP2), cause the majority of Rett syndrome cases [76–78] which is characterized by an apparently normal early development followed by loss of language skill, motor abnormalities, cognitive deficits, stereotyped behavior, respiratory dysrhythmias, and seizures leading sometimes to premature death. *Viaat-Mecp2^−/y^* mice exhibit a significant reduction in amplitude (but not in frequency) of miniature inhibitory postsynaptic currents (mIPSCs) an effect which occurs in the absence of any alteration in amplitude or frequency of miniature excitatory postsynaptic currents (mEPSCs), indicating that MeCP2 deficiency in GABAergic neurons has a cell-autonomous impact on quantal release from glutamatergic neurons [75]. The reduction in GABA content and inhibitory neurotransmission affects synaptic plasticity processes as suggested by the impairment of long-term potentiation (LTP) induced by theta burst stimulation of Schaffer collateral [75]. Previous electrophysiological studies using *Mecp2 *null mice, revealed a significant reduction in spontaneous firing associated with a decrease in amplitude of mEPSCs in layer 5 pyramidal neurons as compared to WT control animals at early presymptomatic and symptomatic stages [79]. In the hippocampus of *Mecp2 *null mice, the diminished level of basal excitatory drive has been shown to contribute, at the network level, to slow down spontaneous rhythmic field potentials activity, generated by the interplay between excitation and inhibition [80]. This condition paradoxically makes the hippocampal network overresponsive to excitatory stimuli. 

An imbalance between excitation and inhibition has been found also in individuals affected by Tuberous sclerosis, a genetic multisystem disorder characterized by widespread hamartomas in several organs, including the brain, heart, skin, eyes, kidney, lung, and liver [81]. Tuberous sclerosis patients exhibit a variety of neurological disorders including epilepsy and autism-like disorders. The affected genes are *Tsc1* and *Tsc2 *encoding hamartin and tuberin, respectively. The hamartin-tuberin complex inhibits the mammalian-target-of-rapamycin pathway that controls cell growth and proliferation [81]. 

Interestingly, a loss of GABAergic function accounts for the hyper excitability observed in an animal model of fragile X syndrome (FXS), a common inherited cause of mental retardation with language deficits, hyperactivity, autistic behavior and seizures. FXS is caused by a trinucleotide expansion of fragile X mental retardation 1 (*fmr1*) gene which prevents the expression of the encoded protein called Fragile X mental retardation protein (FMRP, [82]). As the *Mecp2 *gene, the *fmr1 *gene is located in chromosome X (Xq27.3). The lack of FMRP in animal models of FXS (the *Fmr1*-null mouse) leads to an E/I imbalance in favor of excitation. Among the factors contributing to enhance cell excitability in *Fmr1 *KO animals an impairment of GABAergic circuitry [83] and a decreased expression of GABA_A_ receptor subunits have been reported [84–87]. In subicular neurons, for example, a down regulation of GABA_A_-mediated tonic (but not phasic) inhibition associated with a reduced expression of *α*5 and *δ* GABA_A_ receptors subunits has been found [88]. These alterations may contribute to deficits in cognitive functions and to epileptic activity observed in FXS patients. In contrast, electrophysiological recordings from spiny neurons in the striatum, involved in motor control and in specific aspects of cognition and motivation, have revealed a selective increase in frequency of sIPSCs and mIPSCs, probably secondary to an enhanced probability of transmitter release from GABAergic terminals, suggesting that modifications in GABAergic function in *Fmr1 *KO mice are region-specific [89]. 

Relevant inhibitory synaptic abnormalities (involving both phasic and tonic GABA_A_-mediated inhibition), which may contribute to the abnormal social behavior of *Fmr1 *null mice, are present in the basolateral nucleus of the amygdala [90], which regulates fear and anxiety behaviors.

 Linkage and association studies have revealed that the chromosomal region 15q11-q13 is strongly implicated in ASDs [91]. Maternal duplications of this region remain one of the most common cytogenetic abnormalities found in cases of idiopathic ASDs, which account for 1-2% of cases. Deletion of this region results in either Angelman or Prader-Willi syndrome, depending from which parent the deletion has been inherited [92]. Interestingly, within this chromosomal region, exists a gene cluster of GABA_A_ receptors, *G*abrb*3*, *G*abra*5*, and *G*abrg*3*, encoding for *β*3, *α*5, and *γ*3 subunits, respectively. GABA_A_ receptors are hetero-oligomeric proteins spanning the membrane to form anion-permeable channels. Assembled from eight classes of subunits exhibiting different degrees of homology a large variety of functional receptors with different biophysical and pharmacological properties are expressed in mammalian brain. GABA_A_ receptors play a crucial role in proliferation, migration, and differentiation of precursor cells thus contributing to the establishment of neuronal circuits [93]. A developmental deficit of GABA_A_ receptors function would affect neurogenesis and maturation of neuronal network. Among different GABA_A_ receptor genes, the targeted deletion of *Gabrb3* gene encoding for the *β*3 subunit, which is highly expressed during brain development [94], leads to abnormalities in social behavior, cognitive deficits, motor stereotypes and seizures, reminiscent of the ASDs phenotype [56, 92, 95, 96]. 

Other mutations that affect the GABAergic system concern the homeobox genes* Dlx1 *and *Dlx2*, involved in the development of most telencephalic GABAergic neurons [97]. Interestingly, the human locus with the highest LOD score for autism susceptibility (D2S2188 on chromosome 2q) maps very close to the gene encoding for the GABA synthesized enzyme GAD65 and to *Dlx1 *and *Dlx2.* Furthermore, the autism susceptibility locus D7S477 on chromosome 7q maps within about six megabases of *Dlx5 *and *Dlx6 *which are implicated in the regulation of forebrain GABAergic neurons [98]. This region hosts the gene encoding for Reelin, a protein expressed in cortical GABAergic neurons [99]. Reelin is a signaling protein that plays a pivotal role in the migration of several neuronal cell types and in the development of neuronal connections [100, 101]. Reeler mice, devoid of Reelin, exhibit cytoarchitectonic alterations in their brain similar to those found in autistic patients [102] associated with decrease GABA turnover [103]. 

Interestingly, the removal of the homeobox containing transcription factors Engrailed-2 (*EN2*), known to be involved in the regionalization pattering and neuronal differentiation of the midbrain and hindbrain [104] in mice (*En2−/−mice)* leads to behavioral abnormalities similar to those observed in ASDs patients [105]. In addition, these mice exhibit a reduced expression of parvalbumin and somatostatin positive interneurons in the hippocampus, an effect associated with an increased susceptibility to seizures [105, Table 1].

While the majority of animal models so far examined exhibits a loss of GABAergic function, mice carrying the human R415C mutation in the *Nlgn3 *gene display a gain of function. Neuroligins (NLGs) are specialized cell adhesion molecules that functionally couple the postsynaptic densities with the transmitter release machinery by forming transsynaptic complexes with their presynaptic-binding partners, neurexins [73]. NLG3 R451C KI mice bear a striking phenotype with mimics in many aspects that found in ASDs patients ([106] but see [107]). Functional characterization of these mice has revealed (in contrast with NLG3 KO mice) a loss of NLG3 in the forebrain associated with impaired social interactions and a 50% increase in the frequency of spontaneous inhibitory events with apparent no effects on excitatory synaptic transmission [106]. Interestingly, in NLG3 R451C KI mice, the gain of function is accompanied with a significant increase in the level of the vesicular transporter for GABA, VGAT, and gephyrin, a postsynaptic scaffolding protein, crucial for recruiting and maintaining neurotransmitter receptors in the right place and for ensuring a correct E/I balance. Whether the increased release of GABA selectively affects only a subset of GABAergic interneurons is still unclear. In addition, this animal model exhibits an asymmetric reduction of parvalbumin-positive basket cells across the two hemispheres [108]. However, immunocytochemical data from postmortem material obtained from ASDs patients have revealed an increased density of calbindin-, calretinin-, and parvalbumin-positive interneurons in the hippocampus [23], a condition that would alter neuronal signaling and synchronization leading to cognitive dysfunctions [109]. The enhanced GABAergic innervation may cause a compensatory downregulation of GABA_A_ receptors. The reduction in benzodiazepine-binding sites on GABA_A_ receptors observed in the hippocampus of autistic patients supports this hypothesis [110].

Among autism risk factors, prenatal or neonatal environmental challenges, including early exposure to valproic acid (VPA), a histone deacetylases inhibitor, are widely used as animal models of ASDs [111]. The VPA model has been developed on the basis of the observation that treatment of epilepsy or bipolar disorders in pregnant women (20–24 days after conception) with VPA leads to an increased incidence of ASDs in their children [112]. A unifying hypothesis where the core pathology of the autistic brain consists in hyper-functionality and excessive neuronal processing in local neuronal microcircuits in prefrontal, somatosensory cortex, and amygdala, leading to social and environmental withdrawal has been proposed [113, 114]. Interestingly, as the neuroligin-3 model, the VPA model of ASDs exhibits an asymmetric reduction of parvalbumin-positive cells across the two hemispheres [108]. The disruption of inhibitory circuits may delay critical periods in specific ASDs brain regions [59], thus perturbing *γ*-oscillations implicated in high cognitive functions. 

## 5. Future Perspectives

Although much more work is required to understand the cellular and molecular mechanisms regulating the E/I balance at synapses, it is clear from the reviewed data that GABAergic signaling plays a key role in the construction of neuronal networks and that disruption of GABAergic circuits accounts for several neurodevelopmental disorders including ASDs. A significant progress has been made in characterizing genes involved in synapses formation and maintenance but their role in the organization of neuronal circuits is still limited. From a clinical perspective, a challenged task will be to identify, in animal models of ASDs, the cellular substrates of microcircuits implicated in different cognitive and behavioral deficits associated with ASDs. This can be accomplished by using new optogenetic tools that would allow to selectively activate or silence specific interneuronal populations and to study their functional consequences [116]. With this technique, GFP fusions of channelrhodopsin-related proteins and halorhodopsin, can be delivered into the brain *via *viral infection. In response to different wavelengths of light, label cells and axons can be either depolarized (in the case of channelrhodopsin, [117]) or hyperpolarized (in the case of halorhodopsin), thus allowing to switch on and off selective groups of genetically targeted interneurons and to study the neural basis of different behaviors [118]. This will allow better understanding the mechanistic bases of ASDs and to develop new selectively targeted therapeutic tools for most effective interventions.

## Figures and Tables

**Table 1:** Main alterations of GABAergic signaling present in different animal models of ASDs. For the Rett syndrome, different genotypes are expressed in brackets.

Mouse model	Alterations in GABAergic signaling	Ref.
	Reduced levels of GAD65 and GAD67 (*Viaat-Mecp2^−/y^*)	[75]
	Reduced inhibitory quantal size in layer 2/3 pyramidal neurons of the somatosensory cortex	
*Mecp 2-KO* *(Rett syndrome)*	The E/I balance is shifted to favor inhibition over excitation in cortical networks (*Mecp2^2lox/x^, Nestin-Cre*)	[79]
	Reduced frequency of IPSC-based spontaneous rhythmic field potentials in the hippocampus (*Mecp2^tm1.1Bird^*)	[80]

* Fmr 1-KO * *(X fragile) *	Down regulation of GABAA-mediated tonic inhibition in the subiculum	[88]
	Reduced expression of *α*5 and *δ* GABAA receptor subunits in the subiculum	
	Increased frequency of sIPSCs and mIPSCs in the striatum	[89]
	Reduction in amplitude and frequency of sIPSCs and mIPSCs	[90]
	Reduced GABAA-mediated tonic inhibition	[84–87]
	Reduced GABAergic innervation in the amygdala	
	Reduced expression of GABAA receptor subunits	

*Gabrb 3 KO*	The E/I balance is shifted to favor excitation over inhibition in cortical networks (EEG recordings)	[56]

*Dlx1/Dlx2 KO*	Abnormal cell migration	
	Reduction in the number of GABAergic interneurons in the cortex, olfactory bulb and hippocampus	[97]

*Reln-KO*	Reduced level of GAD67	[103]
	Decreased GABA turnover	

	Reduced expression of parvalbumin- and somatostatin-	
*En2-KO*	positive GABAergic interneurons in the hippocampus	[115]
	Increased susceptibility to seizures	

	Increased frequency of mIPSC	
*Nlg3 R451C KI*	Increased level of VGAT and gephyrin	[106]
	Asymmetric reduction of PV positive basket cells across cortical hemispheres	[108]

*valproic acid *	The E/I balance is shifted to favor excitation over inhibition in the lateral amygdala (multi electrode arrays)	[114]
	Asymmetric reduction of PV positive basket cells across cortical hemispheres	[108]
